# Comparative Properties of Amazonian Oils Obtained by Different Extraction Methods

**DOI:** 10.3390/molecules16075875

**Published:** 2011-07-13

**Authors:** Bianca Silva Ferreira, Camila Guimarães de Almeida, Lara Pereira Faza, Angelina de Almeida, Cláudio Galuppo Diniz, Vânia Lúcia da Silva, Richard Michael Grazul, Mireille Le Hyaric

**Affiliations:** 1 Departamento de Química, Instituto de Ciências Exatas, Universidade Federal de Juiz de Fora, Rua José Lourenço Kelmer, s/n – Campus Universitário, Bairro Martelos, 36036-900, Juiz de Fora-MG, Brazil; 2 Departamento de Parasitologia, Microbiologia e Imunologia, Instituto e Ciências Biológicas, Universidade Federal de Juiz de Fora, 36036-900, Juiz de Fora, Brazil

**Keywords:** antibacterial, antioxidant, cytotoxicity, Amazonian oils, pequi, passion fruit

## Abstract

Pequi (*Caryocar brasiliense Camb.*), babaçu (*Orbignya phalerata Mart.*), buriti (*Mauritia flexuosa*), and passion fruit (*Passiflora edulis*) oils were studied to determine their antibacterial, antioxidant and cytotoxic activities, as well as their total phenol and carotenoid contents. The fatty acid contents were determined by GC-MS. The three types of passion fruit oils studied were refined, cold pressed or extracted from seeds in a Soxhlet apparatus. The oils thus obtained showed differences in antioxidant activity and carotenoid content, but were similar in regard to total phenols. Buriti and pequi had the highest carotenoid contents, while refined and cold pressed passion fruit oil displayed the highest antioxidant activity. Pequi oil was the only oil to display antibacterial and cytotoxic activity.

## 1. Introduction

The Amazon is the world's most important ecosystem, which includes territories belonging to nine South American countries, with 61% being in Brazil. Of the estimated 30 million species of plants found in the Amazon, only a few have been studied and identified so far, based on popular knowledge and scientific studies. Turning these resources into renewable raw materials for industry is a major challenge [1]. Babaçu (*Orbignya phalerata Mart.*), buriti (*Mauritia flexuosa*), and passion fruit (*Passiflora edulis*) are examples of economically important species, whose oils and fats have found various applications in the food, pharmaceutical, cosmetic, and textile industries. As the use of Amazonian vegetable oils has increased in recent years, it is important to aquire a better knowledge of their properties in order to optimize the use of these materials. In this work we studied the antibacterial, antioxidant and cytotoxic activities of pequi (*Caryocar brasiliense Camb*.), babaçu, buriti and passion fruit seed oils. We also determined the β-carotene content and the total phenol contents of each oil. Three types of passion fruit oil were used: industrial refined oil, cold pressed oil and the oil obtained in the laboratory by Soxhlet extraction of the seeds. 

The edible oil and fruits of pequi are used in cooking as well as in traditional medicine for the treatment of colds, coughs, bronchitis, edema and burns [2]. The oil extracted from the nut has applications in soaps and skin emulsions. Babaçu is one of the most important palm trees in Brazil. All the parts of the plants are used, providing food, fuel, fiber, construction materials and more [3]. The edible oil represents 65% of the seed and is used in the fabrication of soaps, surfactants and margarine. Buriti oil is extracted from the fruit of a palm tree native to Brazil. The edible oil, used in cooking, is rich in monounsaturated fatty acids, [4] and natural antioxidants [5]. Buriti oil has found applications in the cosmetic industry due to its emollient properties and can be used as an adjuvant in sun protection [6]. Roots and aerial parts of *Passiflora* species are traditionally used in many countries for its anxiolytic, sedative, diuretic, analgesic, antimicrobial and many other biological effects [7]. Fruits, leaves and seed extracts also display cytotoxic and antioxidant activity, due to the presence of carotenoids, polyphenols and tocopherols [8]. The seed oil, a powerful moisturizing agent, is currently used in many cosmetic formulations.

## 2. Results and Discussion

### 2.1. Physicochemical Analysis

Usual physicochemical parameters of the oils such as density, saponification value (SV), iodine value (IV), peroxide value (PV), acid values (AV), and ester value (EV) were determined (Table 1). The values obtained were consistent with the manufacturer’s analytical reports for the refined oils and with values found in the literature.

The high peroxide (28.63) and acid values (10.86) found for buriti oil indicate that it should not be used for human consumption, as the recommendation for edible oils is a peroxide value less than 10 meq O_2_/kg [14]. When purchased, this oil was stored in a reused polyethylene terephthalate bottle exposed to light, with no indication of the date of manufacture: the prolonged improper handling may have led to lipid peroxidation and partial hydrolysis of the oil.

**Table 1:** Physicochemical analysis.

	Density	SV	IV	PV	AV (% Oleic)	EV (%)
Pequi [9,10]	0.967 ± 0.002	206.8 ± 5.9	50.0 ± 0.93	7.94 ± 0.01	5.4 ± 0.03	97.38 ± 5.9
Babaçu [11]	0.920 ± 0.002	236.9 ± 2.7	18.3 ± 0.50	nd	1.4 ± 0.001	99.41 ± 2.7
Buriti [12]	0.909 ± 0.004	202.4 ± 1.8	61.8 ± 0.49	28.63 ± 0.04	10.9 ± 0.04	87.40 ± 1.9
Passion fruit [13] (Refined)	0.920 ± 0.002	173.4 ± 2.2	236.8 ± 0.46	3.69 ± 0.17	0.2 ± 0.04	99.87 ± 2.3
Passion fruit (Cold pressed)	0.922 ± 0.003	194.3 ± 0.0	109.0 ± 0.52	nd	0.7 ± 0.02	99.62 ± 0.0
Passion fruit (Soxhlet extraction)	0.946 ± 0.002	170.2 ± 2.7	120.3 ± 2,31	nd	1.5 ± 0.03	99.14 ± 2.7

nd: no peroxide was detected.

### 2.2. Fatty Acid Profile

#### 2.2.1. GC-MS

GC-MS analysis was used to estimate the content of the principal fatty acids (unsaturated fatty acids and total saturated fatty acids) of the oils examined in this work. The calculated values were in agreement with the literature data (Table 2). Oleic acid (C18:1) and palmitic acid (C16:0) were the major fatty acids present in pequi and buriti oils. Babaçu oil was the most saturated, with a high content of lauric acid (C12:0; 54.7%). The three passion fruit oils were rich in linoleic acid (C18:2), and contained large amounts of palmitic acid and oleic acid.

**Table 2:** Fatty acids composition (mean percentage) of Amazonian vegetable oils.

	Pequi [10]	Babaçú [11]	Buriti [12]	Passion fruit ^a^ [15]	Passion fruit ^b^ [15]	Passion fruit ^c^ [16]
C6:0	nd	3.3	nd	nd	nd	nd
C8:0	nd	9.2	nd	nd	nd	nd
C10:0	nd	9.6	nd	nd	nd	nd
C12:0	nd	54.7	nd	nd	nd	nd
C14:0	nd	11.8	nd	nd	nd	nd
C16:0	34.5	4.8	16.6	21.6	14.1	12.8
C18:1	65.5	6.5	83.4	21.9	13.2	10.7
C18:2	nd	nd	nd	56.4	72.6	72.6

^a^ Refined oil; ^b^ Cold pressed oil; ^c^ Soxhlet extracted oil; nd = not detected.

### 2.3. Antibacterial Screening

Representative Gram-positive and Gram-negative bacteria were used for the evaluation of antimicrobial activity. The activity was detected by observing and measuring the diameter of microbial growth inhibition zones surrounding the different oils which were added to the test system. The results presented in Table 3 show that only pequi oil displayed antibacterial activity against one of the bacteria (*P. aeruginosa*). The results suggest at least a bacteriostatic activity of pequi oil and its potential use as a preservative. Recent literature reports that oil obtained from *Caryocar coriaceum*, another species of the generus *Caryocar*, shows potential antibacterial activity against *P. aeruginosa*, *S. aureus* and *S. cholerasius* at concentrations of 1.25% [17]. The other oils evaluated in this study did not show any antibacterial activity, being harmless against all the tested bacteria.

### 2.4. Antioxidant Activity

#### 2.4.1. Qualitative Assay

All the oils bleached the DPPH solution (0.2% in MeOH) purple color in the TLC autography qualitative assay, showing that all of them are potential antioxidants.

**Table 3:** Antibacterial activity.

	Inhibition zone (mm)			
	*S. epidermidis*	*S. aureus*	*P. aeruginosa*	*E. coli*
Pequi	-	-	7	-
Babaçu	-	-	-	-
Buriti	-	-	-	-
Passion fruit (Refined)	-	-	-	-
Passion fruit (Cold pressed)	-	-	-	-
Passion fruit (Extracted)	-	-	-	-
DMSO	-	-	-	-

#### 2.4.2. Quantitative Analysis

Quantitative assay revealed varying degrees of antioxidant activity of the oils. Refined and cold pressed passion fruit oil showed the best antioxidant capacity (Table 4). The Soxhlet extract was half as active, probably due to the loss of natural antioxidant compounds caused by heating. Pequi oil was expected to display a higher antioxidant activity, as the pulp contains gallic and quinic acids, powerful antioxidants [18]. Buriti oil, rich in tocopherols and carotenoids13 showed good activity, comparable to refined passion fruit oil. 

**Table 4:** Antioxidant activity.

Oil	EC_50_ (mg/mL)
Pequi	15.54 ± 2.1
Babaçu	70.57 ± 0.4
Buriti	7.70 ± 0.6
Passion fruit (Refined)	5.74 ± 0.1
Passion fruit (Cold pressed)	7.12 ± 0.2
Passion fruit (Soxhlet)	16.84 ± 0,5
Ácido ascórbico (μg/mL)	0.04 ± 0,0

### 2.5. Carotene Determination and Total Phenol Content

The colored buriti and pequi oil are known to contain β-carotene as the main carotenoid in their compositions [19,20]. Pequi, buriti and passion fruit oils were the richest in carotenoids (Table 5), which can be related to their high antioxidant activity. As expected, babaçu oil did not present a high content of carotenoids. Considering the antioxidant activity of the passion fruit oils we were expecting a much higher content of carotenoids in the refined oil. It appeared that it contains less carotenoids than the Soxhlet extract, which also has the highest antioxidant activity. This higher carotene content can be explained by the solvent extraction with an apolar solvent. 

**Table 5:** Total carotene content.

Oil	Carotene content μg/g
Pequi	274.9 ± 3.4
Babaçu	19.8 ± 0.5
Buriti	692.9 ± 6.8
Passion fruit (Refined)	2.3 ± 0.15
Passion fruit (Cold pressed)	4.6 ± 0.1
Passion fruit (Soxhlet)	19.7 ± 0.3

As phenols are also responsible for the antioxidant activity of many other classes as of plants and their extracts [21], we also determined the total phenol contents of each oil (Table 6). The Soxhlet extracted passiflora oil has a lower phenol content than the analogous pressed oil: it is well known that polyphenols are destroyed by heat [22]. The results show that there is not a direct correlation between phenol contents and antioxidant activities. The total phenol content was higher for passion fruit oil which displayed a lower antioxidant activity. Pequi and babaçu oils had the lowest phenol contents, but the highest antioxidant activity. 

**Table 6:** Total phenol content.

Oil	Total phenol content (g/g)
Pequi	229.1 ± 1.65
Babaçu	288.0 ± 1.5
Buriti	309.9 ± 2.9
Passion fruit (Refined)	350.4 ± 1.5
Passion fruit (Cold pressed)	349.6 ± 2.9
Passion fruit (Soxhlet)	339.6 ± 2.8

#### 2.5.1. Cytotoxic Activity

The brine shrimp lethality assay was employed as a toxicity screen. The lethal dose (LD_50_) is the amount of a substance which causes the death of 50% of a group of test organisms. LD_50_ values lower than 1,000 mg/mL indicate toxicity. From the results (Table 7) the tested pequi oil was the only toxic one, suggesting that it should be carefully used. Previous reports have described biological activity and toxicity of leaf extracts of *Caryocar brasiliense* [23,24], but to our knowledge, the toxicity of the oil obtained from the fruit has not been reported yet. Further studies should be performed to determine if the toxicity is specific to the specific sample studied or if it is common to pequi oils.

**Table 7:** Cytotoxic activity.

Oil	LD_50_ (μg/mL)
Buriti	>1000
Pequi	827.62 ± 4,67
Babaçu	>1000
Passion fruit (Refined)	>1000
Passion fruit (Cold pressed)	>1000
Passion fruit (Soxhlet)	>1000

## 3. Materials and Methods

### 3.1. Vegetable Oils

Artisanal pequi, babaçu and buriti oils were purchased from the same producer in a popular market in Januária, Minas Gerais, Brazil. These oils were obtained by cooking the pulp in boiling water, separating the supernatant oil, then drying the oil in a pan until it lost its opacity, and finally filtering. Refined passion fruit oil was purchased from Emfal Empresa Fornecedora de Álcool Ltda, Betim, Minas Gerais, Brazil. Cold pressed passion fruit oil was generously furnished by Extrair, Neale & Reis Indústria e Comércio de Óleos Naturais Ltda, Bom Jesus do Itabapoana – RJ, Brazil.

For the Soxhlet extraction we used seeds from fruits purchased in local markets. The seeds were separated from the pulp in a kitchen food processor, washed with water and frozen until used in order to avoid any modification due to exposure to heat or light. After thawing, the seeds were dried at 40 °C for 2 hours and ground before extraction in a Soxhlet apparatus with petroleum ether for 4 hours. After evaporation of the solvent, a yellow, limpid oil was obtained (yield 13%). The oil was frozen until it was used.

### 3.2. Physicochemical Analysis

The following physicochemical properties of oils were determined: density, saponification value (SV), iodine value (IV), peroxide value (PV), free acid (FA) content, according to AOCS official methods [25]. All analyses were performed in triplicate.

### 3.3. Fatty Acid Profile

The fatty acid profile was determined as fatty acid methyl esters by gas chromatography-mass spectroscopy. The methyl esters were prepared using the AOCS method (AOCS Ce 2-66) [26]. Separation of fatty acid esters was performed on a Shimadzu GC-2010 Gas Chromatograph equipped with a Restek RTX-2330 capillary column (60 m × 0.25 mm × 0.2 mm). The column temperature was programmed at 130 °C for 10 min, then increased to 230 °C at 5 °C/min with a final isothermal period of 13 min. Hydrogen was used as carrier gas with constant linear velocity of 25 cm/sec^−1^. The injector temperature was set at 250 °C, with a split ratio of 1:10. The flame ionization detector temperature was 250 °C. Fatty acid methyl esters (FAMEs) were identified by comparison of retention times with authentic standards (Supelco 37 comp. FAME mix 10 mg/mL in CH_2_Cl_2_), and quantification was performed by the internal normalization method.

### 3.4. Antibacterial Screening

The antibacterial assay was performed using the solid medium diffusion technique [27]. Briefly, from an overnight culture in Tryptic Soy Agar (Himedia, Mumbai, India), 0.5 McFarland bacterial suspensions were obtained and 0.1 mL of each suspension (approximately 10^8^ CFU/mL) was spread on a sterile Mueller-Hinton Agar plate (Himedia, Mumbai, India). After a period of 5-10 min, wells of 5 mm diameter were made in the inoculated Mueller-Hinton plates. Each well was filled with 0.1 mL of a DMSO solution of each tested oil (10 mg/mL). The plates were then aerobically incubated at 35.5 °C for 24 h. The antibacterial activity was determined by measuring the growth inhibition zones recorded in mm in each plate. Nitrofurazone was used as a positive control and DMSO was used as the negative control. The tests were performed in duplicate in which the oils were tested against two Gram positive bacteria strains (*Staphylococcus epidermidis* ATCC12228 and *Staphylococcus aureus* ATCC25923) and two Gram negative bacteria strains (*Escherichia coli* ATTC11229 and *Pseudomonas aeruginosa* ATTC27853).

### 3.5. Antioxidant Activity

#### 3.5.1. Qualitative DPPH Assay

Samples of the different oils were dissolved in dichloromethane and spotted on a thin layer chromatography plate. After total evaporation of the solvent, the plates were sprayed with 2,2-diphenyl-2-picryl-hydrazine (DPPH, Sigma Aldrich, 0.2 mM in methanol). The antioxidant activity was detected after 30 min by the presence of yellow or white spots arising from the reduction of DPPH, against a purple background. β-carotene (0.2 g/L in methanol) was used as positive standard. 

#### 3.5.2. Quantitative Analysis

The antioxidant activity of tested oils was determined using the spectroscopic DPPH free radical scavenging assay [28]. Five concentrations (100, 150, 200, 250 and 300 mg/mL in chloroform) were prepared for each oil, then 0.1 mL of the different mixtures was added to 3.9 mL of a freshly prepared solution of DPPH (0.06 mM in methanol). The absorbance (OD) was measured at 515 nm after 20 min. Ascorbic acid was used as a positive control, DPPH solution without oil solution was used as control and methanol was used as blank. The measurements were performed in triplicate. The antioxidant activity was obtained from the following equation:

Antioxidant activity (%) = [(OD _DPPH_ − OD _oil_) / (OD _DPPH_)] × 100

Percent antioxidant activities were then plotted against concentration. The antioxidant activity of the oils was expressed as EC_50_, defined as the concentration in mg/mL required to scavenge 50% of the DPPH free radical.

### 3.6. Carotene Determination

The quantification was performed by the external standard method, using β-carotene (Sigma-Aldrich) as a reference. The absorbance of the solutions was measured at 436 nm. A solution of β-carotene (0.2 g/L) was prepared in dichloromethane and diluted to obtain final concentrations of 1.26, 2.52, 3.78, 5.04 and 6.30 mg/L which were used for the construction of the standard curve. The concentration of carotenoids in the extract was calculated from the equation straight line, obtained by linear regression.

### 3.7. Total Phenol Content

The total phenol contents in the oils were determined using the Folin-Ciocalteau reagent and gallic acid as standard [29]. The absorbance of the solutions was measured at 765 nm. A solution of gallic acid was prepared by dissolving dry gallic acid (0.5 g) in ethanol (10 mL) and then diluting to 100 mL with water and diluted to obtain phenol concentrations of 0, 50, 100, 150, 250 and 500 mg/L which were used for the construction of the calibration curve. The sample (20 mL) was added to water (1.58 mL), Folin-Ciocalteau reagent (100 mL) and sodium carbonate solution (100 g/L, 300 mL). After 30 min of reaction at room temperature, the absorbance was measured at 765 nm in a Shimadzu 160-UV spectrophotometer. Tests were carried out in triplicate.

### 3.8. Cytotoxicity Screening

The cytoxicity test was performed on the *Artemia nauplii* (brine shrimp larvae) using the Meyer method [30]. The eggs were acquired from an aquarium shop (São Paulo, Brazil) and hatched in artificial sea water, prepared by dissolving NaCl (69 g), MgCl_2_·6H_2_O (59.3 g), Na2SO4 (132 g), CaCl_2_ (6.3 g) and KCl (2.1 g) in distilled water (3 L). After homogenization of the sea water, the pH was adjusted to between 8 and 9 using a Na_2_CO_3_ solution (0.1 M). Brine shrimp eggs were incubated in the artificial seawater at room temperature for 48 hours. An aquarium with a partition was used to allow the migration of the larvae to the lit side. The larvae were fed with biological yeast (18 mg/L in artificial sea water).

The oils were first dissolved in DMSO/Tween (1:1) and the resulting solutions were diluted to obtain the following concentrations: 50 mg/mL, 25 mg/mL, 5 mg/mL, 0.5 mg/mL and 0.05 mg/mL. Aliquots of 0.1 mL of each dilution were added to test tubes containing artificial seawater (4.9 mL) and 10 nauplii. The negative control was carried out in DMSO/Tween (1%) in seawater, and the positive control with K_2_Cr_2_O_7_ (200 μg/mL). Nauplii still alive after 24 h were counted and the lethal concentration (LC_50_) was calculated plotting the percentage mortality versus concentration. All tests were performed in triplicate.

### 3.9. Statistical Analysis

The results presented in this study correspond to the mean of three replicates ± standard deviation. If the variance was homogeneous, the data were assessed by one-way analysis of variance (ANOVA) followed by the Tukey-Kramer test. All calculations were performed using the program Microcal Origin 6.0.

## 4. Conclusions

Among the six Amazonian oils studied in this work, only pequi oil displayed antibacterial and cytotoxic activity. The three passion fruit oils differed in the way they were obtained and also differed in their properties: the refined oil displayed a higher antioxidant activity, but contained fewer carotenoids than the cold pressed oil. The high peroxide and acid contents found for buriti oil points to the lack of quality control in the sail of artisanal products, as this particular oil should not have been commercialized.
